# Translations

**Published:** 1882-01

**Authors:** Seth N. Jordan

**Affiliations:** Columbus, Ga.


### Translations.

BY SETH N. JORDAN, M.D., COLUMBUS, GA.

*From Foreign Journals for the Atlanta Medical Register.*

*Sutures of Nerves and Tendons. H. Kranshold. Centralblatt, No.* 47, 1881.—The case described by Kranshold: A twenty-four year old friseur, who in delirium attempted to cut through both radial arteries, distinguishes itself from other similar cases by the extent of the wound preventing deception as to anastomoses of nerves, and by the final excellent results he obtained. The incision on the left involved the entire breadth of the volar side, taking in the art. rad. and uln., the nervus rad. and uln. entirely, and the nerves median more than three-fourths; further, the tendons of the musc. flex. digi. subl. completely, while the tendons of the m. flex. dig. prof. were partly intact; an analogous injury existed in the right, in the art. rad. uln. and interossei, as well as of the nn. rad., uln, and med. (n. uln. only two-thirds, and stringy) and the tendons mm. flex. dig. subl. and prof., with m. flex. poll. long. After stopping the hemorrhage the author put in on both sides three cat gut sutures through the paraneurotic tissues, first trimming the n. median. He then proceeded to the sutures of the tendons

—four of which on the left and nine on the right—were stitched so that to the support of two cat gut ligatures one carbolized stitch came. Under Lister's antiseptic treatment union by first intention resulted with a return of the sensibility. This latter was entirely restored after three weeks, and thereby flexion and extension, although somewhat modified from the normal, still actively and passively possible. Patient when discharged, was able to perform the same work in hair as before the accident.

---

*The Dihydroxyl Benzoles. By L. Brieger. Zeitschrift f. Klin. Med., III, I,* supported by numerous experiments on animals and man, Brieger recommends the external use alone of hydrochinon (as an anti-fermentive) in those cases, in which, owing to great sensibility, a perfectly non-irritable but strong antiseptic substance is desired. B. recommends hydrochinon in gonorrhœa, but warns against the colored, caustic solutions of this substance. Both resorcin and hydrochinon, Breiger considers unworthy of recommendation, as antipyretica, (after observations on twenty-four cases of typhoid fever). Doses of hydrochinon ranging from 0,4 to 0,6, or hypodermically 0,2 Grm. diminish the temperature in ileo-typhus in the first of the attack; later on, however, up to 1,0 has only a temporary effect. The author noted that the same disadvantage in a similar degree belongs to all of the dihydroxyl benzoles, viz: Sinking of the temperature is rapid, followed by an immediate and sudden rise of same, which latter often exceeds the original temperature. After longer usage the organism does not grow accustomed to these substances, rather the contrary follows—a hypersensitiveness for them, due, the author thinks, to a cumulative effect of the chinon in the system. The author observed in no case a cutting short of the fever. In conclusion, the author emphasizes the parallelism existing between these bodies from a chemical standpoint, and their similar effect phisilogically and therapeutically.

---

*Hypodermic Injections of Iodoform. By Dr. Ed. Thomann Centralblatt f. d. Med. Wissenschaften.*—T. injected subcutaneously iodoform suspended in glycerine, 6.00 : 20.00, and in oil of almonds, 0.30 : 6.00. The injections should be gradually increased, beginning 0,30 and going up to 0.75. T. selected only cases of syphilis of recent infection—the initial sclerosis and pleiades being still present An amelioration of all the symptoms was observable after 10–12 injections. The observation made by Binz and Hogyes, that locally no abscesses appear, our author found verified. After injections of iodoform in glycerine, slight pain followed and disappeared in a short time. The skin becomes red, and on pressure, sensitive. Iodoform in oil produces greater reaction, and the solution thus used must be prepared fresh each time, otherwise it, on standing, becomes brown, after which it is quite irritating, owing to liberation of iodine. Iodine was found in the urine in two hours after the injection. Thomann did not observe any narcotic effect after the largest injection, 0.75. The peculiar iodoform odor was not observable either in the urine, respiration, or perspiration. Temperature and pulse showed no appreciable change after the injections.

---

*On Sutures of Nerves and the Prima Intentie Nervorum. Dissertation. Centralblatt f. Med. Wissenschaften, No.* 45, 1881. —The author communicates the results of numerous experiments carefully made by himself on wounds of nerves for the purpose of establishing the fact of primary reunion, so often claimed of late. He obtained only negative results as far as regards primary union, even in cases where the most favorable conditions were brought to bear. On the other hand, he calls attention to a very fruitful source of error; whenever animals with bisected and sutured ischiadici begin to walk, experimenters so far have regarded this as conclusive evidence of the restoration of the functions of the ischiadici. Our author declares this opinion held by many pathologists to be totally erroneous.

Animals with bisected ischiadici without sutures of the nerves are also soon in a condition to walk and run without a restoration of the funtions of the nerves themselves, but by means of the muscles on the anterior aspect of the thigh, whose innervation is not interrupted. The pretended reunions of bisected and exsected nerves and neuroplastic operations generally are, accordingly to be, at least, more accurately investigated.

---

*Localized Muscular Spasm. E. Remak. Wochenschrift, No.* 20, 1881.—The first case communicated by the author is concerning a man with so-called facial paralysis, which resulted in contractions and co-ordinate movements of the formerly paralyzed muscles. An interesting observation was that the twitchings were synchronous with the closure of the eye-lid, and they ceased when the eye-lids were not moved or when the eyes were closed. A second case, that of a woman fifty-two years old, Remak treated for spasms in the region of the right accessorius and deep muscles of the neck. The positive pole of a moderately strong galvanic current was placed on the right proc. transversi, producing a happy effect. The same procedure had been employed seventeen years before with success by Remak's father on the same patient. The third case was a girl ten years old who was troubled with rythmic twitchings of the muscles of the neck, following a blow. The same treatment used in case No. 2 caused the spasms to entirely disappear.

---

*Connection Between Diseases of the Eye and Ear. Augenheilkunde, LXXXIV. Dransart*—Diseases of the eye are often caused by affections of the ear in a reflex way through the nervus quintus. Dransart saw two cases, in which great deafness followed injuries of the cornea with consequent iritis and formation of posterior synechia, improved after an operation for iredectomy. 3d. case, child with congenital syphilis, and keratitis profunda suffered by means of a snow-ball a trauma of the right eye, which resulted in deafness of the right ear. A young man, who ten years before had lost the power of sight of the right eye and hearing of the right ear in consequence of a blow on the right eye, was robbed of hearing and seeing of the left side, resulting from a fall, striking against the left eye. One patient, who suffered conjunctivitis granulosa heard very imperfectly. This latter condition grew better as soon as his eyes improved. After the operation for ectropium of the right eye, a man of 56 years noticed a marked benefit to his hearing.

---

*Parallelism in the Action of Coniin and Curare. H. Schulz Zeits f. Klin. Med III. p.* 19—Schulz resting his conclusions on experiments made on rabbits, does not consider coniin a poison that physiologically produces convulsions, therein agreeing with Prevost's experiments. Convulsions following the employment of coniin he regards as the expression of beginning suffocation. Only, however, after very large doses do convulsions occur. Therefore Schulz recommends coniin hydrobromatum, a very constant preparation, in all theraputical cases in which curare is indicated, in the place of the latter, especially on account of our ignorance of the efficient alkaloids belonging to curare. Coniin hydrobromatum is readily soluble in water, and is fully equal to curare in fresh solution. This is the only salt of conium that Schulz experimented with to any extent.

---

*Inflammation of the Duct of Stenon. L. Weber. Wochenscrift* No. 33, 1881—A blockage of the duct of Stenon from a plug of fibrin, and an inflammation of the adjacent areolar tissue, in a man 41 years old, produced within a few days, high fever, great swelling, and an erysipelatous redness. The whole process disappeared after Weber succeeded in removing, by pressure, a plug 1½ ctm. long out of the mouth of the duct. Afterwards a quantity of badly smelling pus flowed out of the duct. To remove the induration, W. passed a small sound into the duct for several days. A participation of the parotid was only in so far present, that at the height of the inflammation a stasic swelling of the gland existed.

---

*Nerve Stretching in Ophthalmo-Surgery. L. v. Wecker Augenheilkunde, June,* 1881—v. Wecker performed the operation of stretching the optic nerve on totally blind patients for a relief of pain and halluncinations. He divided the conjunctiva in tangental direction to the medial margin of the cornea, then cutting through the internal rectus, after first passing a suture through its tendon, he separarated Tenon's capsule, and the underlying areolar tissue up to the optic nerve, then the nerve was grasped by means of a tenaculum, and the insertion of the nerve drawn forward. After this, the tenaculum was withdrawn, and the rectus internus stitched to the conjunctiva. An antiseptic bandage was applied. The author promises a further communication regarding the result and indications for the operation.

---

*The Action of Pilocarpine on the Uterus. P. Muller. Dissersertation. Centralblatt, f. Med. Wissenschaften, No.* 23, 1881.—M. considers the pregnant uterus inapplicable for experiments relating to the action of remedies to produce contraction, as uterine contractions are produced too easily, and may follow from other causes. On this account his experiments were made on the puerperal uterus. The results show that injections of pilocarpine produce readily weak contraction, which are of short duration. Of repeated injections, only the first was ever effective. Similar results M. obtained from injections of aq. dest., and consequently he regards the contractions as reflex in their nature. Experiments made to produce abortion were fruitless. The power of pilocarpine to strengthen the contractions is probably to be accepted; its strength is, however, weak.

---

*The Contraction of Blood Vessels on Faradization of the Neck. Katyscherr, Wochenscrift, No.* 5—Under the influence of faradization of the sympathetic in the neck, the inflammatory redness disappears in inflammation of the tympanum in catarrh of the middle ear, and in inflammatory hyperemia following perforations of the tympanum. The tympanum grows either quite pale, or at least looses much of its redness; pain and tinnitus become less, and the hearing itself improves with time. After a sitting lasting from 5 to 15 minutes, this result follows and lasts for several hours. By the application of the constant current, all of these results are less expressed; here interruptions are to be used.

*Cerebral Tumors in Children. A Baginsky. Wochenscrift, No.* 20, 1881—The first case concerns a child 1¼ years old, which grew sick, and after a few months the following status presented: Paralysis of both oculomotorii, complete paralysis of the left abducens, slight of the right, ataxia of left leg; on walking, an inclination to twist to the left side, stupor in a slight degree; infiltration of the apex of the right lung. Under the employment of iodide potassium, extract of malt and cod liver oil, all the symptoms from the side of the brain disappeared, the physical signs in the thorax improved, and in the course of six months the child's general health was exceedingly good. A second case with similar brain symptoms was at the time of the communication, making remarkable progress under the same treatment.

---

*Preparations of Iron Suitable for Hypodermic Medication. G. Neuss, Zeitschrift f. Klin. Med. III, p.* 1.—As an appropriate injection under the skin, Neuss found pyrophosphoricum cum natro citrico (26, 6 per cent iron) in solutions of 1 to 6 part aq. dest, corroborating the observations of M. Rosenthal. Neuss recommends, also, ferrum albuminatum, but adds that it contains less iron and is not so easily preserved in solution.

Ferrum phosphoricum cum ammonio citrico gave rise in one patient of the author's to extreme nervous symptoms, while in two other cases it was well borne.

As inappropriate were demonstrated the following preparations: Ferr. citric oxyd., ferr. oxyd. sacchr. solut., and ferr. oxyd. dialysat. (glycerinatum).

---

*Bromide of Potassium and Injections of Quinine to Prevent "Attacks" in Chronic Insane Persons. B. Kohn, Arch. f. Psych., etc., XI, p* 636.—Attacks of excitement, which often appear in the chronic insane and which are recognized by the patients themselves sometime before appearance by certain prodroma, can be cut short and even prevented by large doses of bromide of potassium. In one case, the history of which the author gives, a complete recovery was obtained by the use of potassio-bromide. The patient had been suffering with the attacks for thirteen years. In another case, also fully stated by the author and similar to the above, a single dose of a solution of quinine 0,8 warmed before injecting subcutaneously, proved successful.

---

*The Action of Oleum Gynocardiae in Skin Diseases. F. J. Pick. Wochenschrift, No.* 3—In the eczema of scrofulous children, chaulmoogra oil aggravates the trouble by causing an artificial eczema. The internal use of it was not conducive of any improvement in the general health. On the other hand, in the chronic form of eczema of adults, a salva of the oil aids much towards diminishing the hypertrophy of the skin. In prurigo and urethritis blennorrhagica, the results were of a negative nature. In three cases of lupus of the hand, the nodules were disintegrated after 3–5 applications. In several cases of lupus of the face, like results were noted.

---

*Bacteria Producing Typhoid Fever. Brautlecht, Virchow's Archives, LXXXIV. p.* 80—B. produced in rabbits a catarrh of the intestinal canal and swelling of Peyer's plaques resembling the typhoid process, by injecting subcutaneously the urine of typhoid patients, and by injecting bacilli reared in culture fluid.

